# Inconsistency in the Standard of Care–Toward Evidence-Based Management of Exertional Heat Stroke

**DOI:** 10.3389/fphys.2019.00108

**Published:** 2019-02-18

**Authors:** Yuri Hosokawa, Takashi Nagata, Manabu Hasegawa

**Affiliations:** ^1^College of Sport and Health Science, Ritsumeikan University, Kusatsu, Japan; ^2^Faculty of Medical Sciences, Kyushu University, Fukuoka, Japan; ^3^Immunization Office, Health Service Division, Health Service Bureau, Ministry of Health, Labour and Welfare, Tokyo, Japan

**Keywords:** rectal thermometry, cold water immersion, pre-hospital care, medical control, exertional heat illness, exertional heat illness treatment

## Abstract

Tokyo 2020 Summer Olympics are projected to experience environmental heat stress that surpasses the environmental conditions observed in the Atlanta (1996), Athens (2004), Beijing (2008), and Rio (2016) Summer Olympics. This raises particular concerns for athletes who will likely to be exposed to extreme heat during the competitions. Therefore, in mass-participation event during warm season, it is vital for the hosting organization to build preparedness and resilience against heat, including appropriate treatment, and management strategies for exertional heat stroke (EHS). However, despite the existing literature regarding the evidence-based management of EHS, rectal thermometry and whole-body cold-water immersion are not readily accepted by medical professionals outside of the sports, and military medicine professionals. Current Japanese medical standard is no exception in falling behind on evidence-based management of EHS. Therefore, the first aim of this paper is to elucidate the inconsistency between the standard of care provided in Japan for EHS and what has been accepted as the gold standard by the scientific literature. The second aim of this paper is to provide optimal EHS management strategies that should be implemented at the Tokyo 2020 Summer Olympics from organizational level to maximize the safety of athletes and to improve organizational resilience to heat. The risk of extreme heat is often neglected until a catastrophic incidence occurs. It is vital for the Japanese medical leadership and athletic communities to re-examine the current EHS management strategies and implement evidence-based countermeasure for EHS to expand the application of scientific knowledge.

## Introduction

Tokyo 2020 Summer Olympics are projected to experience environmental heat stress that surpasses the environmental conditions observed in the Atlanta (1996), Athens (2004), Beijing (2008), and Rio (2016) Summer Olympics (Kakamu et al., 2017). This raises particular concerns for athletes who may have their competitions scheduled during the day time when the exposure to extreme heat is likely. Given that Summer Olympic sports include many outdoor endurance events (e.g., marathon, race walk, and triathlon), it is evident that these athletes may particularly be at risk for exertional heat illness (Bergeron, 2014). Therefore, in mass-participation event during warm season, it is vital for the hosting organization to build preparedness and resilience against heat, including appropriate treatment and prevention strategies for exertional heat illness. According to press releases from Japan Meteorological Agency (2018) and Fire and Disaster Management Agency (2018), Japan experienced the warmest recorded summer in 2018, doubling the number of heat related injury emergency room visits (2018, 7.5 per 10,000 per population; 2015–2017, 4.2 ± 0.2 per 10,000 population) and fatalities (2018, 1.3 per 1,000,000 populations; 2015–2017, 0.6 ± 0.2 per 1,000,000 population) compared to the previous 3-year average. Although these reports do not distinguish exertional heat illness and classic heat illness (i.e., non-exertional), the need to reexamine current medical practice surrounding heat related illness is heightened. In particular, management of exertional heat stroke (EHS), the most severe form of exertional heat illness, should be reviewed by all medical providers since inappropriate care may lead to death when data otherwise suggests 100% survival rate, if treated properly (Demartini et al., 2015). A plethora of studies suggest that determining factor for EHS prognosis is the duration of hyperthermia (≥40°C) incurred by the patient. Therefore, accurate assessment of internal body temperature (e.g., rectal temperature), rapid cooling of the EHS patients using whole-body cold-water immersion, and continuous monitoring of internal body temperature to prevent overcooling are vital in optimizing their survival. Continuous monitoring also helps determine the duration of sustained hyperthermia, which is highly correlated to injury severity (Heled et al., 2004). Despite the existing literature regarding the evidence-based management of EHS, rectal thermometry, and whole-body cold-water immersion are not readily accepted by medical professionals outside of the sports and military medicine professionals. Furthermore, since majority of medical professionals rarely encounter patients who become ill due to over-exertion in the heat, many clinicians have poor understanding of EHS (Casa et al., 2005). Current Japanese medical practice is no exception in falling behind on evidence-based management of EHS (Japanese Association for Acute Medicine, 2015). Therefore, the first aim of this paper is to elucidate the inconsistency between the standard of care provided in Japan for EHS and what has been accepted as the gold standard by the scientific literature. The second aim of this paper is to provide optimal EHS management strategies that should be implemented at the Tokyo 2020 Summer Olympics from organizational level to maximize the safety of athletes and to improve organizational resilience to heat.

## Comparison of Exertional Heat Stroke Triage

Coordination of medical care at athletic events poses unique challenges to the event host and medical providers due to the limited resources available on-site (Adams et al., 2018). Therefore, establishing a evidence-based consensus among medical providers should be of the utmost importance in order to minimize unnecessary hospital transfers and confusion among group of medical providers who are working together for the first time (i.e., volunteers). Scientific literature suggests that the key paradigm for EHS treatment and survival is to *cool first transport second* (Casa et al., 2015). This order of triage may be unique to EHS, as most catastrophic injuries that require medical attention are *transported first*. Consequently, it may be counter-intuitive for many medical providers to treat (i.e., cool) the EHS patient before sending to a hospital, unless an EHS-specific protocol is pre-established (Belval et al., 2018).

In Japan, the paradigm for successful EHS treatment and survival is not well accepted due to two major barriers faced by the medical system. First, in order to perform *cool first* on EHS patients, accurate internal body temperature must be measured rectally to determine the need to cool and the end point of cooling at pre-hospital triage (Casa et al., 2015). However, the current common practice by the emergency medical technician is to use axillary or tympanic membrane temperature (Japanese Association for Acute Medicine, 2015), which provide inaccurate estimate of internal body temperature in exercising individuals (Casa et al., 2007a; Ganio et al., 2009; Taylor et al., 2014; Morán-Navarro et al., 2018). Axillary thermometry is an inexpensive method of internal body temperature assessment and is commonly used to assess pyrexia. Despite its convenience, it has been shown to demonstrate lower value than rectal temperature by mean bias of −0.94 to −1.25°C during indoor exercise in the heat (Ganio et al., 2009). This deviation becomes even larger to a mean bias of −2.07 to −2.58°C when the exercise is conducted outdoors (Casa et al., 2007a). It is evident that extent of bias in axillary temperature to diagnose EHS is too large that it may underestimate the level of hyperthermia and may preclude the patient from receiving the appropriate care. The lower value in axilla than rectal thermometry is likely due to the influence from sweaty skin, which is inherent to someone who has been exercising in thermal environment. Tympanic membrane thermometer is also a common type of thermometer used daily by non-medical and medical personnel. This method is thought to receive less influence from the surface body temperature compared to the axillary method. However, a narrow physical space for measurement (i.e., tympanic membrane), difficulty in capturing the line of sight to the tympanic membrane, influence from earwax buildup, and the estimation of temperature using infrared radiation from the tympanic membrane makes this method vulnerable for invalid measures (Ganio et al., 2009). Consequently, although narrower range than the axillary method, tympanic membrane temperature resulted in a mean bias of −0.67°C and −1.00°C during indoor and outdoor exercise, respectively (Casa et al., 2007a; Ganio et al., 2009). A separate study revealed much narrower range of mean bias (0.10–0.20°C) using a tympanic membrane thermometer during exercise in the heat (Morán-Navarro et al., 2018); however, the exercise-induced hyperthermia observed in this study was moderate (38.3 ± 0.9°C) compared to the previous studies (≈40.0°C), (Casa et al., 2007a; Ganio et al., 2009) suggesting that the deviation in tympanic membrane temperature becomes larger as the internal body temperature increases. Notwithstanding the evidence, the lack of personnel who are trained to take rectal temperature at pre-hospital settings in Japan inevitably delays the EHS diagnosis until the patient is transported to the hospital (Japanese Association for Acute Medicine, 2015). Japanese medical textbooks have also long used the terms heat stroke and heat syncope interchangeably, which also did not help establishing a standardized definition of heat related injuries.

**FIGURE 1:**
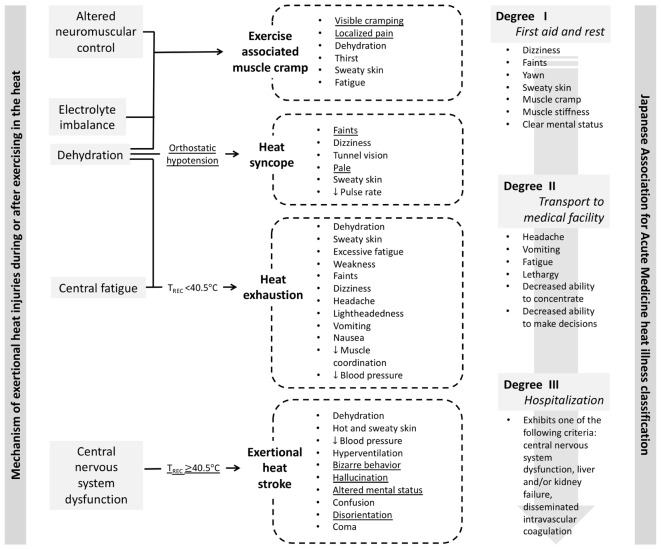
Comparison of the exertional heat illness classification by mechanism (i.e., etiology-based classification; Casa et al., 2015) and by severity of clinical signs and symptoms (Japanese Association for Acute Medicine, 2015). Underlined signs and symptoms may serve as the diagnostic criteria for respective exertional heat illness when the context supports the development of exertional heat illness (i.e., moderate to high environmental condition, physical activity with high metabolic load).

As a result of limited ability to diagnose EHS based on rectal temperature and lack of standardized definition to describe heat related injuries Yasuoka et al. (1999), proposed the assessment of heat illness by the severity of signs and symptoms that is classified into Degree I (mild), II (moderate), and III (severe). This classification was readily adopted by the Japanese emergency medical community since similar severity category was used to describe burn injuries and heat failure. Degree I heat illness is considered benign and self-resolving with rest in cool area, external body cooling, oral rehydration, and sodium supplementation. Clinical symptoms classified under Degree I heat illness include dizziness, faints, yawn, sweaty skin, muscle cramp, muscle stiffness, and clear mental status. Degree II heat illness is considered more severe than Degree I, and its clinical signs and symptoms include headache, vomiting, fatigue, lethargy, decreased ability to concentrate, and deceased ability to make decisions. Japanese Association for Acute Medicine (JAAM) suggests that patients with Degree II heat illness should be transported to medical facility to restore normal body temperature and hydration status (Japanese Association for Acute Medicine, 2015). The most severe form of heat illness is classified as Degree III, which requires hospitalization. The clinical manifestation of Degree III heat illness includes central nervous dysfunction, liver and kidney failure, and disseminated intravascular coagulation (DIC). The heat illness severity classification was later validated by Okudera et al. (2002) by retrospectively examining the medical data from patients who were admitted the emergency department due to heat illness. It is important to note that organ failures and DIC are the outcomes of EHS that were *not* treated properly (Casa et al., 2015; Stearns et al., 2016), and they can be prevented if early recognition of hyperthermia and aggressive cooling are implemented (Casa et al., 2007b). In other words, unless the pre-hospital medical standards for EHS changes to allow rectal thermometry and cooling before transport, the classification system is opening a room for EHS prognosis that may be debilitating or in the worst case, fatal. Figure 1 compares the exertional heat illness classification by mechanism (i.e., etiology-based classification) and by severity of clinical signs and symptoms (i.e., JAAM classification). While mechanism based-classification allows clinicians to look for presence (or absence of) distinct clinical presentations, symptom-based classification without rectal thermometry is subjective, which opens a window for overlooking EHS (Figure 1).

**FIGURE 2:**
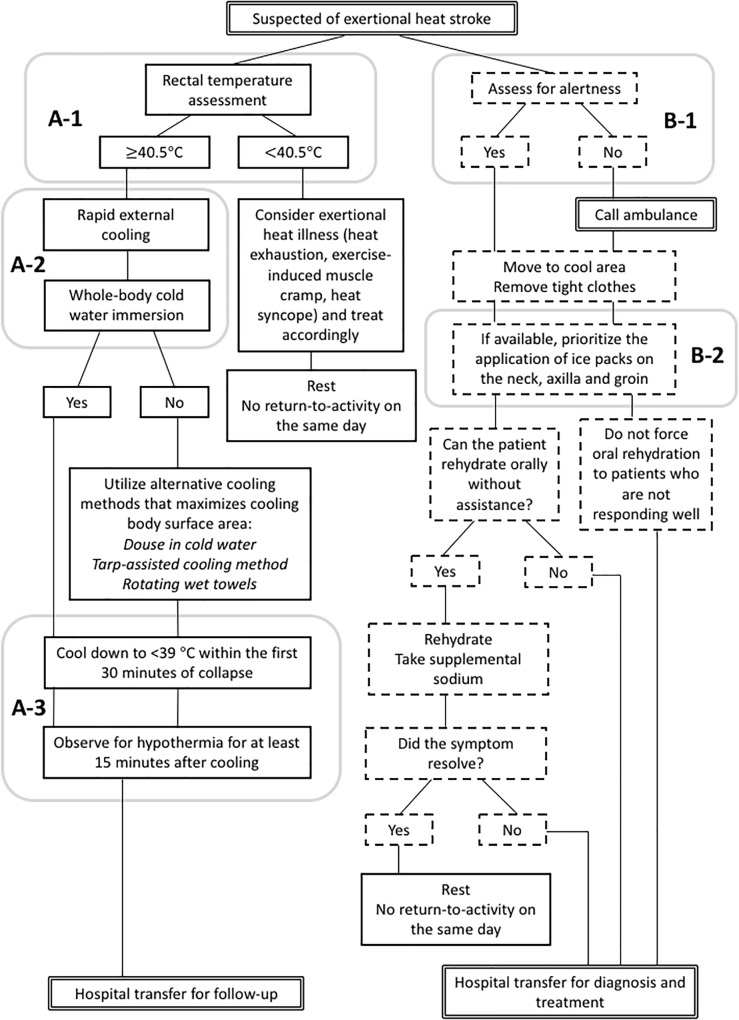
Comparison of exertional heat stroke (EHS) triage flow charts between the accepted gold-standard by the current scientific literature (**A**, left; Belval et al., 2018) and the current Japanese medical standard allowed by domestic medical regulations (**B**, right; Japanese Association for Acute Medicine, 2015). **(A-1)**, EHS is diagnosed with rectal thermometry; **(A-2)**, primary method of cooling is whole-body cold-water immersion; and **(A-3)**, rectal temperature is monitored continuously to determine the end point for the treatment. **(B-1)**, EHS is diagnosed by clinical sign (i.e., alertness) and **(B-2)**, on-site cooling is optional and the methods are partial-body cooling.

The second barrier to *cool first transport second* comes from the hesitancy among medical professionals to cool EHS patients using whole-body cold-water immersion due to the fear of inducing shock (Japanese Association for Acute Medicine, 2015). Although JAAM recognizes the importance of aggressive cooling in their Heat Illness Treatment Guideline 2015 (Japanese Association for Acute Medicine, 2015), it does not outline the specific method for cold water-immersion. Instead, clinicians choose to apply ice packs on major arteries and use alcohol wipes to induce evaporative cooling for EHS patients, both of which have demonstrated inadequate cooling rates for EHS treatment (Casa et al., 2007b). Literature suggest that cooling modality for EHS treatment should have a cooling rate of 0.15°C min^−1^, which can be achieved using cold water-immersion of 1∼20°C (Casa et al., 2007b; McDermott et al., 2009). Other methods such as ice packs covering the body (≈0.03°C min^−1^) and fanning (≈0.05°C min^−1^) have very limited ability to cool exercise-induced hyperthermia in a timely manner. Since the severity of organ damage and chance of survival are dictated by the duration of sustained internal body temperature above 40°C (Casa et al., 2007b), the ability to cool the patient before transport will directly impact the chance of survival from EHS. Inconvenience from cold water immersion (i.e., wet skin, limited access to supplemental treatments) should not outweigh the need to protect organs from sustained hyperthermia (Casa et al., 2007b). Although the chance is very unlikely, cardiopulmonary arrest case that warrant automated external defibrillator (AED) application would be managed, similarly regardless of the use of cold water immersion; patent should be removed to a dry area, have the contact area for AED pads dried, and promptly begin cardiopulmonary resuscitation and apply AED (Casa et al., 2007b). Figure 2 compares the common paths of EHS triage with and without the rectal thermometry and whole-body cold-water immersion as accepted methods.

## Exertional Heat Stroke Management Strategies for the Tokyo 2020 Summer Olympics

Recent documents (Japan Medical Association, 2015; Matsumoto, 2018) have reiterated the importance of the use of rectal thermometry and cold water immersion for EHS diagnosis and treatment; however, due to the aforementioned barriers, they have yet to become the standard of care in Japan. One of the solutions to overcome these barriers before the Tokyo 2020 Summer Olympics is to implement a medical control system that allow trained professionals (e.g., physician, nurse, emergency medical technician, and athletic trainer) to implement an EHS specific pre-hospital care, allowing the recognition and treatment of EHS *before* arriving to the hospital (Belval et al., 2018). Since the number of medical professionals who are trained to use of rectal thermometry and cold water immersion for EHS patients is currently limited, the Summer Olympic Games can turn into a great opportunity for Japanese medical professionals to receive training in these methods and support a paradigm shift in the outdated EHS diagnosis and treatment methods used in Japan. In the context of mass sporting, establishing the chain of commands ahead of the time to coordinate not only the on-site medical personnel but also the local hospital network, event officials, and security becomes vital in ensuring that all parties involved in the care of the collapsed athlete are following the same protocol (Adams et al., 2018).

In addition to the proper pre-hospital care for EHS, there should be pre-established criteria to modify, discontinue, or cancel competitions to safeguard athletes especially with the growing prevalence of extreme heat days exceeding =28°C in wet bulb globe temperature (WBGT) (American College of Sports Medicine Armstrong et al., 2007). Since external factors, such as the number of preliminary matches that must be completed within the fixed number of days, sponsorship, and primetime hours for broadcasting will likely influence the competition hours for televised sporting events, lack of flexibility to adjust the competition time freely will greatly affect the ability to make modifications to the existing schedule. Nevertheless, event organizer should strive to avoid the time of extreme heat according to the historical climate data. Furthermore, there should be an action plan for environmental heat hazards that outlines (1) the upper WBGT threshold to modify, discontinue or cancel events, (2) the chain of command about who makes the final decision to modify, discontinue or cancel events, and (3) specific actions that need to be followed once an event is modified, discontinued, or canceled. Similar to the medical control system for EHS pre-hospital care, emergency action plan for environmental heat hazard should be shared among all parties involved in the event. Moreover, simulating the worst case possible will help elucidate the extent (or lack) of resources available to handle such an emergency, and will facilitate discussions to improve organizational resilience to heat by addressing specific scenarios of anticipated heat hazards (Adams et al., 2018).

## Conclusion

In conclusion, an evidence-based countermeasure for EHS has not been implemented in Japan and strong leaderships from medical and athletic communities are warranted to reduce the risk of EHS at the Tokyo 2020 Summer Olympics. The need to implement evidence-based management of EHS has long been overlooked due to the unfamiliarity of the condition by most healthcare providers. It is critical that medical community recognize the shortcoming of their current practice and implement what is proven to be best in the current literature.

## Author Contributions

YH created the main conceptual ideas for the paper. All authors conducted a thorough review of the existing literature and contributed to the manuscript writing and review process.

## Conflict of Interest Statement

The authors declare that the research was conducted in the absence of any commercial or financial relationships that could be construed as a potential conflict of interest.
